# Health behaviors of caregivers of childhood cancer survivors: a cross-sectional study

**DOI:** 10.1186/s12885-020-06765-w

**Published:** 2020-04-07

**Authors:** In Young Cho, Nack-Gyun Chung, Hee Jo Baek, Ji Won Lee, Ki Woong Sung, Dong Wook Shin, Jung Eun Yoo, Yun-Mi Song

**Affiliations:** 1 Department of Family Medicine, Kangbuk Samsung Hospital, Sungkyunkwan University School of Medicine, Seoul, Republic of Korea; 2 Department of Pediatrics, College of Medicine, The Catholic University of Korea, Seoul, Republic of Korea; 3 Department of Pediatrics, Chonnam National University Hwasun Hospital, Chonnam National University Medical School, Gwangju, Republic of Korea; 4 Department of Pediatrics, Samsung Medical Center, Sungkyunkwan University School of Medicine, Seoul, Republic of Korea; 5 Department of Family Medicine, Samsung Medical Center, Sungkyunkwan University School of Medicine, 81 Irwon-Ro, Gangnam-gu, Seoul, 06351 Republic of Korea; 6 Department of Family Medicine, Healthcare System Gangnam Center, Seoul National University Hospital, Seoul, Republic of Korea

**Keywords:** Cancer survivors, Child, Caregivers, Health behavior, Alcohol drinking, Exercise, Smoking

## Abstract

**Background:**

Caregiving for childhood cancer survivors may be burdensome for caregivers and affect their physical health and health behaviors. However, studies examining health behaviors in caregivers of childhood cancer survivors are scarce. This study aimed to examine health behaviors of caregivers of childhood cancer survivors by comparing them with those of the general population, and analyze associated factors.

**Methods:**

This study included 326 caregivers of childhood cancer survivors recruited from 3 major hospitals in South Korea and 1304 controls from the Korean National Health and Nutritional Examination Survey matched for age, sex, and education level. We compared health behaviors between the two groups by using conditional logistic regression analyses, and investigated factors associated with unhealthy behaviors in caregivers by using multiple logistic regression analyses.

**Results:**

Caregivers were less likely to be physically inactive (aOR: 0.69, 95% CI: 0.51, 0.92) compared to controls, and this was more evident in women (aOR: 0.65, 95% CI: 0.45, 0.94). However, caregivers were more likely to be binge drinkers (aOR: 2.26, 95% CI: 1.73, 2.97), especially if they were men (aOR: 13.59, 95% CI: 8.09, 22.82). Factors associated with unhealthy behaviors in caregivers differed by the type of behavior. Current smoking risk was lower in female caregivers and in those with more comorbidities. Increasing age, female sex, higher education level, and lower household income were associated with lower risk of binge drinking. Higher household income and anxiety were associated with lower risk of physical inactivity, while depression was associated with higher risk of physical inactivity.

**Conclusions:**

Caregivers of childhood cancer survivors were more likely to engage in binge drinking, but less likely to be physically inactive. Strategies to promote adherence to desirable health behaviors in caregivers are needed with consideration of their socioeconomic and clinical factors, such as number of comorbidities.

## Background

The number of childhood cancer survivors is increasing continuously due to improving survival rates of childhood cancer [1]. In the United States, 5-year survival rates for childhood cancer have improved from 58% for cases diagnosed from 1975 to 1979, to 84% for cases diagnosed from 2008 to 2014 [2]. Meanwhile, childhood cancer survivors may require support and care from informal caregivers, which is usually their parent or guardian; and caregivers may be affected negatively by their caregiving experience [3, 4]. Previous studies have demonstrated that caregivers tend to feel unhealthy [5], depressed [6], or have impaired quality of life [7]. Caregiving may also affect the physical health of family caregivers, which may in turn influence the health outcomes of the children being cared for, and form a vicious cycle [8, 9].

In a study of self-reported medical morbidity in cancer caregivers, the most commonly reported morbidities were hypertension, high cholesterol, chronic back pain and heart disease [10]. Therefore, carrying out healthy lifestyle behaviors that are closely related with these diseases may exert positive effects on caregivers’ health. Smoking, along with excessive alcohol consumption and physical inactivity are well-known modifiable risk factors for cardiovascular disease, hypertension and dyslipidemia [11, 12]. Since parents whose children are diagnosed with cancer face a major transitional stage in their lives and experience stress due to their caregiving role, practice of health behaviors may have been affected [13, 14]. Unhealthy behaviors could deteriorate the health of caregivers as well as the childhood cancer survivors being cared for, and therefore research on caregivers’ health behaviors is of great importance.

Previous studies on childhood cancer caregivers have mainly examined psychological outcomes or quality of life [3, 4, 15–17]. Few studies have examined health behaviors in cancer patient caregivers [18], and those examining caregivers of childhood cancer survivors are even scarcer. A previous study conducted in the US examined health behaviors in caregivers of childhood cancer survivors and noted that only one third met national guidelines for physical activity, and 6.3% were current smokers [19]. However, no study was found examining health behaviors of caregivers of childhood cancer survivors in an Asian population. In addition, no study has compared health behaviors of caregivers of childhood cancer survivors with the general population.

Therefore, this study was conducted to examine and compare the health behaviors of caregivers of childhood cancer survivors with the general population, and to investigate factors associated with unhealthy behaviors in caregivers.

## Methods

### Study subjects

Study subjects were the caregivers of childhood cancer survivors recruited at 3 major hospitals in South Korea, as part of an ongoing cohort of childhood cancer survivors and their caregivers to evaluate their long-term health problems. Childhood cancer survivors were those diagnosed with any childhood cancer before they were 19 years old, and had completed all treatment for cancer, including chemotherapy, surgery, radiotherapy, and hematopoietic stem cell transplant. Neonates and those who were treated for recurred or metastatic cancer were excluded from the study. Among a total of 367 caregivers recruited from October 2017 to December 2018, we excluded those without responses for smoking (*n* = 9), physical activity (*n* = 5), drinking (*n* = 23), education level (*n* = 1), or income (*n* = 5). Thus, 326 caregivers of 240 childhood cancer survivors were finally included in the analysis. Because both the mother and the father of a child could participate in the study, there could be more than one caregiver per child.

Control subjects were selected from the participants of the Korean National Health and Nutritional Examination Survey conducted between the years 2016 and 2017 (KNHANES VII). Every caregiver was matched to four controls of the same age, sex, and education level, which resulted in a total of 1304 controls. Although we could not select controls based on their children’s characteristics because of lack of detailed information on offspring in KNHANES VII, we put an effort to include those who were likely to have children by excluding men who had never married and women who had never given birth.

This study was approved by the institutional review boards (IRB) of each institute (2017–08-024 at Samsung Medical Center (SMC), KC17ONDI0694 at the Catholic University of Korea Seoul St. Mary’s Hospital, and CNUHH-2017-159 at Chonnam National University Hospital), and was performed in accordance with the Declaration of Helsinki. All caregivers included in the study provided informed consent. The IRB at SMC waived the need for informed consent from the control group, because they had already provided informed consent for the KNHANES and the dataset is open for public use, and it does not include any individually identifiable information.

### Health behaviors

We collected information on caregivers’ demographic characteristics (age, sex, education, monthly household income), comorbidities (hypertension, diabetes mellitus, dyslipidemia, cardiovascular disease, and cancer), and health behaviors (cigarette smoking, alcohol consumption, and physical activity) by using a self-administered questionnaire. We also asked whether they were the main caregiver for the childhood cancer survivor. We collected information on the childhood cancer survivors including the child’s age, type of cancer, the type of treatment they had received, and the date of end of treatment through review of medical records.

Health behaviors were classified into the following unhealthy behaviors: (1) current smoking, where those who smoked daily or sometimes smoked were considered current smokers and those who had never smoked or had quit smoking were considered non-smokers, (2) binge drinking, where women who drank 5 drinks or more per occasion and men who drank 7 drinks or more per occasion were considered binge drinkers, (3) physical inactivity, where those who exercised less than 3 times per week were considered to be physically inactive.

### Statistical analysis

We compared characteristics between the caregiver and control groups matched for age, sex, and education level, by using chi-square tests for categorical variables, and Student’s t-tests for continuous variables. We estimated odds ratios (OR) and 95% confidence intervals (CI) for unhealthy behaviors (current smoking, binge drinking, and physical inactivity) of childhood cancer caregivers compared to controls by using conditional logistic regression analysis, and also obtained adjusted odds ratios (aOR) by adjusting additionally for income, number of comorbidities, and cancer diagnosis.

We evaluated factors associated with unhealthy behaviors in caregivers by using multiple logistic regression analyses. We also examined factors associated with engaging in 2 or more unhealthy behaviors. All statistical analyses were carried out using Stata 14.0 (Stata Corp, Texas, USA), and two-sided *p*-values of < 0.05 were considered statistically significant.

## Results

The caregivers included in this study were the parents of 240 childhood cancer survivors. The mean age of childhood cancer survivors at recruitment was 13.7 (standard deviation (SD): 6.0) years, and mean time lapse after completing cancer treatment was 4.7 (SD: 3.9) years. Around half of the childhood cancer survivors being cared for had been treated for a hematologic malignancy (47.5%), while the other half had been treated for a solid tumor (52.5%). All childhood cancer survivors had been treated with chemotherapy, 45.0% had received surgery, 35.4% radiation therapy and 22.5% hematopoietic stem cell transplant (Supplementary Table 1, Additional File 1).

Table 1 shows the distribution of the demographic and clinical characteristics of caregivers and controls. The majority of caregivers were women (62.9%) and well-educated (65.0% possessing a college degree or above). The majority did not have any comorbidity (80.7%), while 3.7% had been previously diagnosed with cancer. The majority were also the main caregiver (67.8%), of which most were female (84.2%). Caregivers were also more likely to have lower household income compared with the control group (*p* < 0.001).

**Table 1:** Comparisons of general characteristics between caregivers of childhood cancer survivors and controls

	Caregivers (***N*** = 326)	Controls (***N*** = 1304)	***p***-value*
**Age at recruitment, mean (SD), years**	44.1 (6.2)	44.1 (6.2)	–
**Sex**			
Male	121 (37.1)	484 (37.1)	–
Female	205 (62.9)	820 (62.9)	
**Education level**			
High school graduate or below	114 (35.0)	456 (35.0)	–
College graduate or above	212 (65.0)	848 (65.0)	
**Monthly household income, won**			
< 2 million	38 (11.7)	91 (7.0)	< 0.001
2~4 million	127 (39.0)	323 (24.8)	
4~6 million	112 (34.4)	400 (30.7)	
> 6 million	49 (15.0)	490 (37.6)	
**Number of comorbidities**^a^			
None	263 (80.7)	1106 (84.8)	0.159
One	45 (13.8)	134 (10.3)	
Two or more	18 (5.5)	64 (4.9)	
**Previous cancer diagnosis**	12 (3.7)	49 (3.8)	0.948
**Main caregiver status**			
Yes	221 (67.8)	NA	–
No	103 (31.6)	NA	–
Not reported	2 (0.6)	NA	–
**Depression**^b^			
Normal	131 (40.3)	NA	–
Borderline depression	118 (36.3)	NA	–
Depression	76 (23.4)	NA	–
**Anxiety**^b^			
Normal	183 (56.7)	NA	–
Borderline anxiety	106 (32.8)	NA	–
Anxiety	34 (10.5)	NA	–

Data are presented as number (percentages), or as mean (standard deviations) where specified

*N* Number of subjects, *SD* Standard deviation, *NA* Not available

*Obtained by Student’s t-tests for continuous variables and Pearson’s chi-square tests for categorical variables

^a^Includes diabetes mellitus, hypertension, dyslipidemia, or cardiovascular diseases

^b^Depression and anxiety were defined by the Hospital Anxiety and Depression Scale scores. Cutoff values were < 8 for normal, 8–10 for borderline, and > 10 for both depression and anxiety

Table 2 shows the health behaviors of caregivers of childhood cancer survivors compared to those of controls. Overall, caregivers were more likely to be binge drinkers (aOR: 2.26, 95% CI: 1.73, 2.97) and less likely to be physically inactive (aOR: 0.69, 95% CI: 0.51, 0.92). Stratification by sex showed that, in men, caregivers were more likely to engage in binge drinking (aOR: 13.59, 95% CI: 8.09, 22.82), while caregivers were less likely to be physically inactive (aOR: 0.65, 95% CI: 0.45, 0.94) in women.

**Table 2:** Odds ratio (95% confidence intervals)^a^ for health behaviors of caregivers of childhood cancer survivors as compared to the health behaviors of controls

	Controls	Caregivers	*p*-value*	Unadjusted analysis		Multivariable adjusted analysis	
	N (%)	N (%)		OR (95% CI)	*p*-value	aOR (95% CI) †	*p*-value
**All subjects**							
Current smoking	239 (18.3)	49 (15.0)	0.163	0.76 (0.53,1.09)	0.134	0.79 (0.54,1.13)	0.197
Binge drinking	256 (19.6)	120 (36.8)	< 0.001	2.38 (1.83,3.10)	< 0.001	2.26 (1.73,2.97)	< 0.001
Physical inactivity	992 (76.1)	239 (73.3)	0.300	0.86 (0.65,1.14)	0.297	0.69 (0.51,0.92)	0.013
**Male**							
Current smoking	197 (40.7)	42 (34.7)	0.228	0.77 (0.53, 1.17)	0.222	0.73 (0.47,1.13)	0.161
Binge drinking	48 (9.9)	71 (58.7)	< 0.001	13.41 (8.16, 22.04)	< 0.001	13.59 (8.09,22.82)	< 0.001
Physical inactivity	358 (74.0)	89 (73.6)	0.926	0.98 (0.62, 1.55)	0.925	0.76 (0.47,1.24)	0.269
**Female**							
Current smoking	42 (5.1)	7 (3.4)	0.305	0.65 (0.29,1.48)	0.306	0.56 (0.24,1.29)	0.172
Binge drinking	208 (25.4)	49 (23.9)	0.665	0.92 (0.65,1.32)	0.665	0.87 (0.61,1.26)	0.474
Physical inactivity	634 (77.3)	150 (73.2)	0.211	0.80 (0.56,1.13)	0.208	0.65 (0.45,0.94)	0.022

Binge drinking: ≥5 drinks per occasion for females and ≥ 7 drinks per occasion for males. Physical inactivity: exercise < 3 times per week

*N* Number, *OR* Odds ratio, *aOR* Adjusted odds ratio, *CI* Confidence intervals

*obtained by Pearson’s chi-square tests

†adjusted for income, number of comorbidities (diabetes mellitus, hypertension, dyslipidemia, or cardiovascular disease), and previous cancer diagnosis

^a^estimated by conditional logistic regression after matching for age, sex, and education level

Table 3 shows the factors associated with unhealthy behaviors in caregivers. Female sex and increasing number of comorbidities were associated with lower risk of current smoking. Increasing age, female sex, and higher level of education were associated with lower risk of binge drinking, while higher household income was associated with higher risk of binge drinking. Higher household income and anxiety were associated with lower risk of physical inactivity, while depression was associated with higher risk of physical inactivity. Increasing age, female sex, and possessing a college or a higher degree were all associated with lower risk of engaging in 2 or more unhealthy behaviors.

**Table 3:** Factors associated with unhealthy behaviors^a^ in caregivers of childhood cancer survivors

	Current smoking		Binge drinking		Physical inactivity		≥2 unhealthy behaviors^e^	
	OR (95% CI)^b^	***p***-value*	OR (95% CI)^b^	***p***-value*	OR (95% CI)^b^	***p***-value*	OR (95% CI)^b^	***p***-value*
**Age, 1-year increase**	0.99 (0.92, 1.06)	0.689	0.92 (0.87, 0.97)	0.001	0.99 (0.94, 1.04)	0.702	0.93 (0.88, 0.98)	0.011
**Sex**								
Male	1 (ref)	< 0.001	1 (ref)	< 0.001	1 (ref)	0.564	1 (ref)	< 0.001
Female	0.05 (0.02, 0.15)		0.21 (0.10, 0.42)		1.24 (0.60, 2.55)		0.20 (0.10, 0.40)	
**Education level**								
High school graduate or below	1 (ref)	0.167	1 (ref)	0.045	1 (ref)	0.831	1 (ref)	0.003
College graduate or above	0.55 (0.24, 1.28)		0.55 (0.31, 0.99)		1.07 (0.60, 1.91)		0.39 (0.21, 0.73)	
**Monthly household income**								
< 2 million won	1 (ref)	0.063	1 (ref)	0.017	1 (ref)	0.009	1 (ref)	0.656
2~4 million won	0.53 (0.16, 1.73)		1.02 (0.41, 2.53)		0.74 (0.28, 1.97)		0.87 (0.35, 2.14)	
4~6 million won	0.28 (0.08, 1.02)		1.71 (0.67, 4.35)		0.70 (0.25, 1.90)		1.09 (0.42, 2.78)	
> 6 million won	0.22 (0.04, 1.07)		2.60 (0.91, 7.44)		0.25 (0.08, 0.74)		0.63 (0.20, 1.95)	
**Number of comorbidities**^c^								
None	1 (ref)	0.006	1 (ref)	0.773	1 (ref)	0.389	1 (ref)	0.601
One	0.44 (0.14, 1.34)		1.05 (0.49, 2.21)		0.86 (0.40, 1.82)		0.64 (0.28, 1.47)	
Two or more	0.20 (0.02, 1.73)		1.26 (0.39, 4.04)		3.51 (0.82, 15.00)		1.09 (0.33, 3.56)	
**Previous history of cancer**	0.99 (0.10, 9.60)	0.994	0.16 (0.02, 1.43)	0.102	0.79 (0.18, 3.46)	0.754	0.47 (0.08, 2.61)	0.387
**Main caregiver**								
Yes	1 (ref)		1 (ref)		1 (ref)		1 (ref)	
No	1.44 (0.59, 3.54)	0.420	1.48 (0.75, 2.91)	0.257	1.13 (0.53, 2.40)	0.744	1.85 (0.92, 3.70)	0.083
**Years after end of treatment, 1-year increase**	0.93 (0.83, 1.05)	0.242	1.05 (0.98, 1.14)	0.160	0.97 (0.90, 1.05)	0.471	1.00 (0.92, 1.08)	0.973
**Depression, yes**^d^	1.49 (0.65, 3.42)	0.350	0.73 (0.41, 1.30)	0.290	3.57 (1.95, 6.57)	< 0.001	1.28 (0.70, 2.35)	0.418
**Anxiety, yes**^d^	0.57 (0.24, 1.36)	0.206	1.21 (0.68, 2.15)	0.524	0.50 (0.27, 0.92)	0.027	0.74 (0.40, 1.36)	0.336

*OR* Odds ratio, *CI* Confidence intervals

*p-value or *p* for trend if applicable (ordinal variable with more than two categories)

^a^Binge drinking: ≥5 drinks per occasion for females and ≥ 7 drinks per occasion for males. Physical inactivity: exercise < 3 times per week

^b^Multiple logistic regression was performed including all variables in the table

^c^Comorbidities include diabetes mellitus, hypertension, dyslipidemia, and cardiovascular disease

^d^Depression and anxiety were assessed through the Hospital Anxiety and Depression Scale scores. Cutoff values were < 8 for normal (reference group), and ≥ 8 for cases of depression and anxiety

^e^ ≥ 2 unhealthy behaviors refers to engaging in at least 2 of the 3 unhealthy behaviors evaluated in our study (current smoking, binge drinking, and physical inactivity)

## Discussion

This was the first study to compare health behaviors between caregivers of childhood cancer survivors and the general population. This study showed that caregivers were more likely to be binge drinkers, especially in men, but less likely to be physically inactive, especially in women. Furthermore, this study found that age, sex, socioeconomic factors (such as education and income), and clinical characteristics (such as number of comorbidities) were the factors associated with unhealthy behaviors in caregivers, although the associations differed by the type of unhealthy behavior.

Although the current smoking rate was slightly lower in caregivers than in the general population, it is noticeable that the smoking rate in caregivers was suboptimal (15.0%), and much higher than the rate reported among American childhood cancer caregivers (6.3%) [19]. This discrepancy may be a reflection of the smoking rate of the general population, since recent statistics have shown that the overall adult smoking rate was 17.5% in Korea, and 10.5% in the US [20]. However, this may also mean that public health education may be needed to further reduce smoking rate in caregivers of childhood cancer survivors. It is important that parents refrain from smoking, especially if their children are diagnosed with cancer. Smoking is an important risk factor for cancer, and childhood cancer survivors are at increased risk for subsequent neoplasms [21]. Furthermore, in a study assessing risk factors for smoking among adolescent survivors of childhood cancer, exposure to other smokers increased the likelihood of smoking, and the authors recommended providing smoking cessation programs targeted to family members to prevent smoking in adolescent childhood cancer survivors [22]. When we further investigated factors associated with smoking in caregivers, female sex and higher number of comorbidities were associated with lower odds of smoking, suggesting that men and those without comorbid diseases should be targeted for education on smoking cessation.

Physical inactivity was less prevalent among caregivers than in the general population, especially in female caregivers. However, physical activity levels were still suboptimal. Adequate physical activity is important for both childhood cancer survivors and their caregivers. Regular physical activity would be beneficial for caregivers through its preventive effect on chronic diseases including hypertension, dyslipidemia, and diabetes. A study on exercise interventions using pedometers for parents of children with cancer also showed mental health benefits [23]. Furthermore, a study showed that more active mothers have more active children [24], and a systematic review on the effects of exercise-based interventions for cancer survivors demonstrated that exercise can reduce fatigue and impact positively on quality of life [25]. Research on childhood cancer survivors have reported an 8.2-fold increased risk of mortality due to cardiovascular diseases [26], and primary prevention through physical activity may help reduce this risk and improve overall health [27]. In our study, physical inactivity was inversely associated with monthly household income, supporting the theory that lack of time and money are common barriers for caregivers’ health promoting behaviors [28]. Given the benefits of physical activity, strategies to make caregivers practice adequate physical activity irrespective of household income levels are needed.

Interestingly, while depression was associated with higher risk of physical inactivity, anxiety was associated with lower risk of physical inactivity. Physical activity has previously been shown to be associated with lower depressive symptoms, while exercise has been suggested to improve depression [29]. However, it is not clear how anxiety may be associated with increased physical activity. Most studies have found that anxiety is associated with decreased physical activity [30]. However, other studies have reported that although depressive subjects tended to have decreased physical activity, anxious subjects did not [31, 32]. Another study showed that anxious subjects were more physically active, and suggested that anxiety could increase restlessness, or anxious individuals could have used physical activity as a coping mechanism [33]. Therefore, anxiety could also be associated with increased physical activity in caregivers, but further studies may be needed to determine how this association could be present in caregivers.

It is noticeable that binge drinking was markedly prevalent among male childhood cancer caregivers, and the risk of binge drinking was around 13.6 times higher in male childhood cancer caregivers compared with the male general population. When we further investigated factors associated with binge drinking in caregivers, older age and higher education level were found to be protective against binge drinking. This is in agreement with general population-based studies, where lower education level and younger age were associated with harmful alcohol use [34, 35].

Interestingly, we found that higher monthly household income was associated with higher risk of binge drinking in our study. Studies on the relation between income level and binge drinking have shown inconsistent findings. Some studies have shown that subjects with lower income engage in risky drinking behavior more often than those with high incomes [36, 37]. Other studies conducted in the general Korean population have found no association between income and harmful alcohol use [34, 35]. However, a Canadian study on factors associated with risky single occasion drinking also found that subjects with the highest income had higher odds of risky drinking behavior. Furthermore, previous research examining the relationship between alcohol use and earnings demonstrated that drinkers earn more than non-drinkers, but those who drink excessively earn less, implying an inverse u-shape association between alcohol use and income [38].

This study has several limitations. First, information was collected using self-reported questionnaires, which may cause information bias due to underreporting of unhealthy behaviors. Second, this study was conducted and designed as a cross-sectional study, and behavioral changes of caregivers could not be compared before and after the diagnosis of cancer. Third, we were unable to exclude those who might be caregivers of childhood cancer survivors from the control groups, because family history of cancer was not available in the KNHANES VII data. Fourth, the findings of our study may not be applicable to the general population of childhood cancer caregivers, because study participants were recruited in only 3 major hospitals in Korea. Furthermore, our study was conducted limitedly in caregivers of childhood cancer survivors. Therefore, findings from our study may not be generalizable to caregivers of childhood cancer patients currently undergoing treatment. In addition, findings of our study results may also not be applicable to other countries because of differences in culture or medical systems.

Despite these limitations, our study has unique strengths. First, it is the first to compare health behaviors of caregivers of childhood cancer survivors with the general population. Second, we were able to consider a wide range of variables, including the characteristics of childhood cancer survivors verified through revision of medical records.

## Conclusion

Caregivers were less likely to be physically inactive compared with the general population, but more likely to be binge drinkers. Considering the importance of practicing healthy lifestyle behaviors for both childhood cancer survivors and their caregivers, strategies to promote adherence to desirable health behaviors in childhood cancer caregivers are necessary with consideration of their socioeconomic and clinical factors.

## Supplementary information

**Additional file 1 Table S1.** Characteristics of childhood cancer survivors.

## Data Availability

The datasets generated and/or analyzed during the current study are not publicly available due to information that could compromise research participant privacy or consent.

## Abbreviations

IRB: Institutional Review Board
KNHANES: Korea National Health and Nutritional Examination Survey
OR: Odds ratio
aOR: Adjusted odds ratio
CI: Confidence intervals
SD: Standard deviation
n: Number of subjects
CTx: Chemotherapy
N/A: Not available
