# Supplementary material for: Health behaviors of caregivers of childhood cancer survivors: a cross-sectional study
Source: BMC Cancer. 2020 Apr 7;20:296. doi: 10.1186/s12885-020-06765-w (PMC7140330; doi:10.1186/s12885-020-06765-w)
Supplement: Supplementary file 1 — Additional file 1 Table S1. Characteristics of childhood cancer survivors. [file 12885_2020_6765_MOESM1_ESM.docx]

**Table S1.** Characteristics of childhood cancer survivors

|  | **N = 240** |
| --- | --- |
| **Age at recruitment, mean (SD), years** | 13.7 (6.0) |
| **Tumor type** |  |
| Solid | 126 (52.5) |
| Hematologic | 114 (47.5) |
| **Treatment received** |  |
| Chemotherapy | 240 (100.0) |
| Chemotherapy + Surgery | 108 (45.0) |
| Chemotherapy + Radiation therapy | 85 (35.4) |
| Chemotherapy + HSCT | 54 (22.5) |
| **Time lapse since treatment completion, mean (SD), years** | 4.7 (3.9) |

*N*, number; *SD*, standard deviation; *HSCT*, Hematopoietic stem cell transplant

Data are presented as number (percentages), or as mean (standard deviations) where specified.
